# Detection of human herpesviruses in the cerebrospinal fluid from patients diagnosed with or suspected of having progressive multifocal leukoencephalopathy

**DOI:** 10.1186/1471-2377-13-200

**Published:** 2013-12-13

**Authors:** Kazuo Nakamichi, Naoki Inoue, Toshio Shimokawa, Ichiro Kurane, Chang-Kweng Lim, Masayuki Saijo

**Affiliations:** 1 Department of Virology 1, National Institute of Infectious Diseases, Toyama 1-23-1, Shinjuku-ku, Tokyo 162-8640, Japan; 2 Department of Ecosocial System Engineering, Interdisciplinary Graduate School of Medicine and Engineering, University of Yamanashi, Takeda 4-3-11, Kofu City 400-8511, Yamanashi, Japan

**Keywords:** Cerebrospinal fluid, Human herpesvirus, JC virus, Progressive multifocal leukoencephalopathy, Quantitative PCR testing

## Abstract

**Background:**

Progressive multifocal leukoencephalopathy (PML), a fatal demyelinating disease caused by JC virus (JCV), occurs mainly in immunocompromised patients. While JCV DNA is detected in the cerebrospinal fluid (CSF) from a certain proportion of patients suspected of having PML, JCV-negative patients may also develop brain lesions due to other infectious agents. This study assessed the prevalence of six herpesviruses in the CSF from patients diagnosed with or suspected of PML.

**Methods:**

Two hundred and ninety-nine CSF specimens and clinical data were collected from 255 patients, including 31 confirmed PML cases. Quantitative PCR assays were carried out to detect the genomic DNA of JCV, herpes simplex virus (HSV), varicella-zoster virus (VZV), cytomegalovirus (CMV), Epstein-Barr virus (EBV), and human herpesvirus 6 (HHV-6).

**Results:**

Herpesvirus DNAs were detected in the CSF specimens from 29 of 255 patients (11.4%). HSV-1 and CMV were detected in JCV-negative patients, whereas VZV and EBV were detected in both CSF JCV-positive and -negative individuals. The herpesvirus-positive patients had underlying disorders that caused immunosuppression, such as HIV infection, congenital immunodeficiencies, and hematologic malignancies, and presented with neurologic symptoms and MRI lesions, mainly in the cerebral white matter. The median values of CSF cell counts and protein levels in the herpesvirus-positive patients were slightly higher than those in the PML patients.

**Conclusions:**

The results demonstrate that herpesviruses are occasionally detected in the CSF from PML patients and immunocompromised individuals suspected of having PML. Thus, this study provides a significant basis for the diagnosis and treatment of neurological disorders in immunocompromised patients.

## Background

Progressive multifocal leukoencephalopathy (PML) is a rare but fatal demyelinating disease of the central nervous system (CNS) caused by JC virus (JCV), a small DNA virus belonging to the family *Polyomaviridae*, genus *Polyomavirus*[1-3]. Humans are infected with JCV asymptomatically during childhood and are persistently infected with it throughout life. From 50–90% of adults have been reported to be serologically positive for JCV [1-4]. In some severely immunocompromised patients, JCV activates and causes a lytic infection in the oligodendrocytes, leading to PML [1-4]. Although PML is mainly diagnosed in patients with HIV-infection, it is also observed in patients with immunodeficiency due to a hematological malignancy, chemotherapy, transplantation, lymphocyte depletion, or autoimmune disorders, such as systemic lupus erythematosus, and in those under treatment with immunosuppressive agents [1,3,5]. In addition, PML has recently been identified in patients receiving immunomodulatory therapies with monoclonal antibodies, such as natalizumab, rituximab, and efalizumab [1-3,6].

The detection of JCV DNA in the cerebrospinal fluid (CSF) by PCR is a reliable and less-invasive diagnostic marker of PML, particularly when combined with typical magnetic resonance imaging (MRI) patterns [1,7]. CSF testing for JCV DNA using a quantitative PCR technique has become the current diagnostic standard [6]. In Japan, real-time PCR testing for JCV DNA in CSF specimens has been partly supported by the Laboratory of Neurovirology, Department of Virology 1, National Institute of Infectious Diseases (NIID), Tokyo, Japan, since 2007. The CSF from approximately 11% of patients (48 of 419) was found to be positive for JCV DNA and these patients were diagnosed with PML [8]. However, no JCV could be detected in the CSF samples from the remaining approximately 89% of patients, implying that a large proportion of these subjects might have developed brain disorders due to other infectious or non-infectious causes.

Herpesviruses, in particular, herpes simplex virus (HSV), varicella-zoster virus (VZV), cytomegalovirus (CMV), Epstein-Barr virus (EBV), or human herpesvirus 6 (HHV-6), are major etiological agents of encephalitis and other CNS infections in immunocompromised persons [9-13]. This study sought to assess whether these herpesviruses contribute to the CNS involvement in patients diagnosed with or suspected of having PML.

## Methods

### Collection of CSF specimens and clinical data

The study was conducted under the approval from the Ethical Committee for Biomedical Science in the NIID (approval number 339). Informed consent from patients or their family members was also obtained. Patients suspected of having PML on the basis of neurological symptoms and/or MRI patterns were enrolled in this study. Upon request to the patients’ physicians, CSF testing for JCVDNA was routinely performed regardless of patient age, gender, underlying disease, or medical history. Two hundred and ninety-nine CSF specimens were collected by lumbar puncture from 255 patients from April 2007 to the end of January 2010, immediately frozen, and then transferred to the NIID for PCR testing. For 44 of the patients, CSF testing was repeated during their follow-up period. Clinical data were collected from the patients’ physicians through standardized questionnaires. The following data were analyzed: age, gender, underlying diseases, manifestations of neurologic symptoms, pattern of brain MRI lesions, and CSF leukocyte counts and total protein levels.

### Real-time PCR testing for viral DNA in CSF specimens

Total DNAs were extracted from the CSF specimens using a QIAamp DNA Blood Mini Kit (Qiagen, Valencia, CA) and then used as PCR templates as described previously [8,14,15]. Since most of the CSF specimens had been frozen at the hospitals, they also contained cellular components. Quantitative real-time PCR assays targeting the DNAs of JCV [8,14,15], VZV [16], CMV [17], and HHV-6 [17] were carried out as described in the earlier reports. HSV-1/2 and EBV DNAs were quantified using an artus HSV-1/2 LC PCR Kit (Qiagen) and LightCycler EBV Quantification Kit (Roche, Penzberg, Germany), respectively, according to the protocols supplied by the manufacturers.

### Statistical analysis

The detection rates of herpesvirus DNAs in the CSF specimens and the sex ratios of patients in each group were statistically compared by means of a two-tailed Fisher’s exact test. For multiple testing, the resulting *P*-value was corrected using the Benjamini-Hochberg method. Differences in the ages and CSF cell counts and protein contents between patient groups were compared by nonparametric analyses using the Mann–Whitney *U* test. All *P*-values less than 0.05 were judged to be statistically significant.

## Results

### Detection of JCV in CSF from patients suspected of having PML

The study population comprised 166 males and 89 females. The mean age of the subjects was 56.3 years (median 59.0 years, range 4–89 years, SD = 18.0), excluding 1 male patient whose age was not definitely stated. The underlying diseases of 255 subjects were as follows: HIV infection (*n* = 52, 50 males, 2 females), hematologic disorders (*n* = 51, 39 males, 12 females), autoimmune disorders (*n* = 33, 10 males, 23 females), other diseases (*n* = 46, 29 males, 17 females), and unknown (*n* = 73, 38 males, 35 females). A total of 299 CSF specimens from 255 patients were subjected to the real-time PCR assay for JCV DNA, and 42 samples (14%) were found to be positive for JCV DNA. The median JCV load in these specimens was 3.2 × 10^4^ copies/mL (range 1.5 × 10^2^ – 4.8 × 10^8^ copies/mL, SD = 7.8 × 10^7^). The prevalence of JCV DNA and underlying diseases in the patient population are shown in Table 1. Thirty-one of 255 patients (12%) were diagnosed with PML based on clinical findings and JCV DNA-positive CSF. These PML patients had HIV infection (10 patients, 19%), hematologic disorders (13 patients, 26%), autoimmune disorders (3 patients, 9%), or other diseases (5 patients, 11%). No JCV DNA was detected in the CSF specimens from 73 patients who had no clinically apparent underlying disorders.

**Table 1:** Prevalence of CSF JCV DNA and underlying diseases in the patient population

	**No. (%) of patients**		
	**Total**	**JCV-positive**	
**Underlying disease**	**( *n * = 255)**	**( *n * = 31)**	
HIV infection	52	10	(19.2)
Hematologic disease	51	13	(25.5)
Autoimmune disease	33	3	(9.1)
Other disease	46	5	(10.9)
Unknown	73	0	(0)

### Detection of herpesvirus DNA in CSF from patients diagnosed with or suspected of having PML

**Figure 1:**
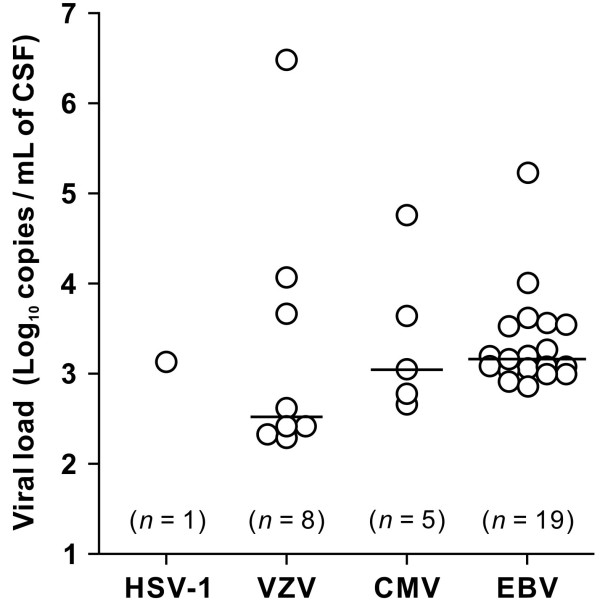
**Viral loads of herpesvirus DNAs in the CSF specimens from patients.** The CSF viral loads of HSV-1, VZV, CMV, and EBV are shown (left to right). Each open circle indicates the copy number of viral DNA in each sample, and the horizontal lines represent the medians.

The next series of analyses were conducted to clarify the etiological contribution of herpesviruses to CNS disease by the detection of herpesvirus DNA in 299 CSF specimens from 255 patients diagnosed with or suspected of having PML. Among the 299 CSF samples, 31 were positive for herpesvirus DNA (Table 2). HSV-1, VZV, CMV, and EBV were detected in 1 (0.3%), 8 (2.7%), 5 (1.7%), and 19 (6.4%) specimens, respectively. Two specimens were positive for CMV and either HSV-1 or EBV. No amplification signal was observed for HSV-2 and HHV-6 in any sample. HSV-1 and CMV were detected only in JCV-negative CSF specimens. In contrast, VZV and EBV were detected in both JCV-positive and -negative samples. The viral DNA level of HSV-1 in 1 specimen was 1.3 × 10^3^ copies/mL. The median viral loads of VZV, CMV, and EBV were 3.3 × 10^2^, 1.1 × 10^3^, and 1.5 × 10^3^ copies/mL, respectively (Figure 1). Although the DNA levels of these viruses in most specimens ranged from 10^2^ to 10^4^ copies/mL, more than 10^4^ copies/mL of VZV, CMV, and EBV DNAs were found in some samples.

**Table 2:** Number of herpesvirus DNA-positive and -negative CSF specimens

	**No. (%) of CSF specimens**		
**Herpesvirus**	**Total**^**a**^	**JCV-positive**	**JCV-negative**
**DNA**	**(*n*** **= 299)**	**(*n*** **= 42)**	**(*n*** **= 257)**
HSV-1	1 (0.3)	0 (0)	1 (0.4)
HSV-2	0 (0)	0 (0)	0 (0)
VZV	8 (2.7)	1 (2.4)	7 (2.7)
CMV	5 (1.7)	0 (0)	5 (1.9)
HHV-6	0 (0)	0 (0)	0 (0)
EBV	19 (6.4)	5 (11.9)	14 (5.4)

^a^Two specimens were positive for CMV and either HSV-1 or EBV DNA.

### Proportion of patients for whom CSF specimens were herpesvirus DNA positive

Table 3 shows the numbers and proportion of patients for whom the CSF specimens were positive for herpesvirus DNA. Among the 255 subjects, CSF herpesvirus DNA was detected in 29 (11%). HSV-1 and CMV were detected in CSF JCV-negative patients, while VZV and EBV were detected in JCV-negative and positive-patients. The detection rate of herpesvirus DNAs in JCV-positive patients (19%) was higher than that in JCV-negative individuals (10%), but this difference was not statistically significant. VZV was detected in 8 patients, from one of whom EBV-positive CSF specimens were obtained during the follow-up. Five patients were found to be positive for CMV, and HSV-1 and EBV were concomitantly detected in CMV-positive samples from 1 patient each. Sixteen individuals were found to be positive for EBV but not for other herpesviruses. The EBV-positive rate appeared to be higher than those of other herpesviruses. In addition, the detection rate of EBV in JCV-positive patients was statistically higher than that in JCV-negative patients (*P* = 0.032).

**Table 3:** Number of patients in whom herpesvirus DNA was detected in the CSF

	**No. (%) of patients**		
**Herpesvirus**	**Total**	**CSF JCV-positive**	**CSF JCV-negative**
**DNA in CSF**	**(*n*** **= 255)**	**(*n*** **= 31)**	**(*n*** **= 224)**
Total	29 (11.4)	6 (19.4)	23 (10.3)
HSV-1 and CMV	1 (0.4)	0 (0)	1 (0.4)
VZV	7 (2.7)	1 (3.2)	6 (2.7)
VZV or EBV^a^	1 (0.4)	0 (0)	1 (0.4)
CMV	3 (1.2)	0 (0)	3 (1.3)
CMV and EBV	1 (0.4)	0 (0)	1 (0.4)
EBV	16 (6.3)	5 (16.1)	11 (4.9)

^a^VZV and EBV DNA was detected in different CSF specimens collected from the same patient during the follow-up period.

### Underlying diseases of herpesvirus DNA-positive patients

The clinical data of 29 herpesvirus DNA-positive patients were analyzed. The patients comprised 23 males and 6 females. The mean age of all except 1 patient was 52.5 years (median 53.5 years, range 30–84 years, SD = 14.7). There were no statistically significant differences in the age and sex ratios between the herpesvirus DNA-positive and -negative patients. The underlying diseases of the patients who provided herpesvirus DNA-positive CSF specimens are summarized in Table 4. Of the 29 patients, 27 (93%) were found to have underlying disorders that may cause immunosuppression. Sixteen patients (55%) had HIV infection, and the severe loss of peripheral blood CD4-positive T cells was observed in most cases. VZV, CMV, and/or EBV were detected in CSF from the HIV-positive patients. Eight patients (28%) suffered from hematologic diseases, such as non-Hodgkin’s lymphoma and aplastic anemia, and had been treated with hematopoietic stem cell transplantation, combination chemotherapy, or other immunosuppressive drugs. Three patients (10%) had other underlying diseases, such as lupus nephritis, chronic renal failure, or primary angiitis of the CNS, and received immunosuppressive therapy. Among the subjects in each group, the proportion of the herpesvirus DNA-positive patients with HIV infection (31%) and that with hematologic diseases (16%) were statistically higher than that with other underlying diseases (4%), as compared by multiple statistical testing (*P* = 0.000 and 0.048, respectively). In addition, both JCV and herpesvirus DNAs were detected in the CSF specimens from 6 patients with either HIV infection or hematologic diseases.

**Table 4:** Underlying diseases of patients in whom herpesvirus DNA was detected in the CSF

**Herpesvirus**	**No. of patients**	**Underlying disease (%)**							
**DNA in CSF**		**HIV infection**		**Hematologic disease**		**Other disease**^**a**^		**Unknown**	
Total	29	16	(55.2)	8	(27.6)	3	(10.3)	2	(6.9)
HSV-1 and CMV	1	0	(0)	1	(100)	0	(0)	0	(0)
VZV	7	2^b^	(28.6)	3	(42.9)	2	(28.6)	0	(0)
VZV or EBV	1	1	(100)	0	(0)	0	(0)	0	(0)
CMV	3	3	(100)	0	(0)	0	(0)	0	(0)
CMV and EBV	1	0	(0)	0	(0)	1	(100)	0	(0)
EBV	16	10^c^	(62.5)	4^d^	(25.0)	0	(0)	2	(12.5)

^a^The data include patients with autoimmune disorders.

^b^One patient was positive for JCV.

^c,d^The results include 2 and 3 JCV-positive patients, respectively.

### Clinical features of herpesvirus DNA-positive patients

**Figure 2:**
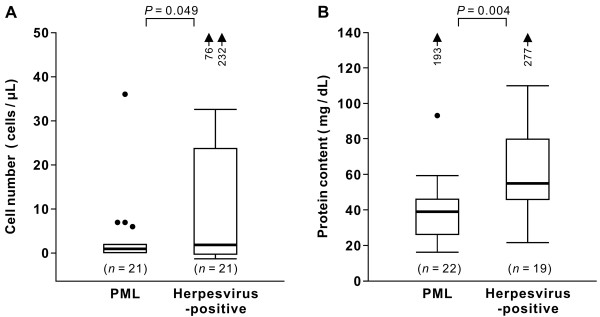
**CSF cell counts (A) and total protein contents (B) in PML and herpesvirus-positive patients.** Since the CSF cell counts and/or total protein contents were not defined in the questionnaires in some cases, the numbers of patients are not identical in panels **A** and **B**. The data of patients positive for both CSF JCV and herpesvirus DNAs are not included in each panel. In box-and-whisker plots, the thick horizontal line within each box is the median; the lower and upper boundaries are the 25th and 75th percentiles, respectively; vertical whiskers extend over the range; and dots and arrows show outliers.

In the final set of analyses, the clinical features of the patients with herpesvirus-positive CSF were compared to those of PML patients. Table 5 shows the appearance patterns of neurologic symptoms and brain lesions. In the study population, 25 patients were positive for JCV but provided herpesvirus-negative CSF specimens. These PML patients presented with diverse neurologic symptoms, such as paralysis, dementia, dysarthria, dysphagia, and/or visual impairment (data not shown). MRI lesions were found mainly in the cerebral white matter (CWM) (84%), and a smaller proportion of patients showed lesions in other sites, such as the cerebellum (16%) and brain stem (28%). Among the 24 patients in whom herpesviruses were detected in the CSF, 20 individuals (83%) had neurologic manifestations. MRI lesions were identified in the CWM (75%), cerebellum (29%), brain stem (8%), and other sites (13%). There were no statistically significant differences in the proportion of individuals with lesions at each site between the PML and herpesvirus-positive patients. The VZV- or EBV-positive patients displayed lesions not only in the CWM, but also in other areas of the brain. In contrast, the lesions were localized in the CWM in the HSV-1- and/or CMV-positive patients. Figure 2 shows the results of CSF cell counts and total protein contents of the PML and CSF herpesvirus-positive patients. Since the CSF cell counts and/or total protein contents were not defined in the questionnaires in some cases, the numbers of patients are not identical in Figure 2A and 2B. In both patient groups, the median values of cell counts for both mono- and polynuclear cells (Figure 2A) and protein contents (Figure 2B) were at normal or near-normal levels. However, a considerable proportion of herpesvirus-positive patients exhibited higher cell numbers and protein levels when compared to those of PML patients, and these differences were statistically significant (*P* = 0.049 and 0.004, respectively).

**Table 5:** Neurologic symptoms and brain MRI patterns in the PML and herpesvirus-positive patient groups

			**MRI(T2/FLAIR) lesion (%)**^**a**^				
**Detected**	**No. of patients**	**Neurologic symptom (%)**	**Cerebral white matter**				
**Viral DNA in CSF**				**Cerebellum**	**Brain stem**	**Other**	**Unknown**
JCV^b^	25	25 (100)	21 (84.0)	4 (16.0)	7 (28.0)	2 (8.0)	2 (8.0)
Herpesviruses^c^	24	20 (83.3)	18 (75.0)	7 (29.2)	2 (8.3)	3 (12.5)	1 (4.2)
HSV-1 and CMV	1	1 (100)	1 (100)	0 (0)	0 (0)	0 (0)	0 (0)
VZV	6	6 (100)	5 (83.3)	1 (16.7)	1 (16.7)	0 (0)	1 (16.7)
VZV or EBV	1	1 (100)	1 (100)	1 (100)	0 (0)	1 (100)	0 (0)
CMV	3	2 (66.7)	3 (100)	0 (0)	0 (0)	0 (0)	0 (0)
CMV and EBV	1	1 (100)	1 (100)	0 (0)	0 (0)	0 (0)	0 (0)
EBV	12	9 (75.0)	7 (58.3)	5 (41.7)	1 (8.3)	2 (16.7)	0 (0)

^a^The data include patients with multiple lesion sites.

^b^The patients were negative for herpesvirus DNA in their CSF.

^c^The patients were negative for JCV DNA in their CSF.

## Discussion

The present study aimed to comprehensively assess the prevalence of six human herpesviruses in the CSF specimens from patients diagnosed with or suspected of having PML. Since the aim of this study was the detection of herpesviruses DNA using PCR from the CSF samples of the patients diagnosed with or suspected PML associated with several immuno-suppressive underlying diseases, the detection of virus DNA indicates the existence of these viruses, but the data do not always indicate the main contribution to brain damage.

One of the most important findings is that EBV was present in the CSF of approximately 16% of the confirmed PML patients. Although the detection of EBV as well as JCV in the CSF has been reported previously [18-20], the prevalence of EBV in the CSF specimens from the relatively large number of patients suspected of having PML has not been determined previously. This data suggest that it is not rare to concomitantly detect JCV and EBV in PML cases. Although the EBV-positive patients had not been diagnosed with EBV-related diseases when the specimens were collected, it would be interesting to see whether these patients later developed EBV-associated neurological disorders. It would be attractive to hypothesize that EBV infection is involved in the progression of PML progression as the detection rate of EBV in the CSF from JCV-positive patients was higher than that in JCV-negative patients. However, this result might be due to differences in the proportion of immunocompromised individuals between the JCV-negative and -positive populations. VZV was detected in the CSF from one PML patient with AIDS (CD4 cell count, 8 cells/μl). The detection rate of VZV appeared to be lower than that of EBV, and the co-detection of JCV and VZV in CSF was reported in one previous report [20]. It is possible that this patient developed both PML and VZV encephalitis.

It is of interest to note that HSV-1/2, CMV, and HHV-6 were not detected in the CSF specimens from any PML patient. Two previous reports demonstrated that JCV was concomitantly detected with CMV or HSV-1 in the CSF [20,21]. The data obtained in this study indicate that the CSF from some PML patients was HSV-1 and/or CMV DNA-positive, although the prevalence was low. Another important finding in this study is that HSV-1, VZV, CMV, and EBV were detected in more than 10% of the CSF specimens from patients suspected of having but not diagnosed with PML. Based on neurological symptoms, MRI lesion patterns, and underlying disease, it seems reasonable that these patients were suspected of having PML. A large proportion of the patients that were found to be positive for herpesviruses had HIV infection or hematologic disorders, suggesting that there is a significant relationship between the presence of herpesviruses in the CSF and severe immunosuppression due to AIDS, chemotherapy, or hematopoietic stem cell transplantation. As the distribution of CSF cell numbers and protein contents partially overlapped in the PML and herpesvirus-positive patients, these parameters may not directly contribute to a diagnosis of PML. It is likely that the inflammatory response was inhibited under the immunosuppressive conditions in both patient groups. However, it is worth focusing on the significant proportion of the herpesvirus-positive patients showing high CSF cell counts. In these patients, it can be postulated that the brain inflammation was induced by the lytic herpesvirus infection. In such a situation, the amount of herpesvirus DNA might be increased by the migration of the infected cells into the CSF, as the PCR assays were performed using total DNA extracted from frozen CSF.

In some herpesvirus-positive cases, a combination of two herpesviruses, such as CMV and HSV-1, CMV and EBV, and VZV and EBV, were detected in the CSF, which is consistent with previous reports describing the co-detection of herpesviruses in CSF [18,20,22,23]. It was also observed that VZV and EBV were detected in different CSF specimens from one patient during the follow-up period. This patient presented with lesions in the CWM and basal ganglia, at which time VZV was detected. At repeat CSF testing 3 months later, EBV, but not VZV, was detected in the CSF, and lesions were identified in the CWM and cerebellum. This observation indicates that although VZV propagation in the CNS was reduced, EBV infection or reactivation occurred during the follow-up period in this patient.

Currently, no specific treatment has been established for PML. Restoration of the immune system, either by combination antiretroviral therapy for patients with AIDS or by moderating the immunosuppressive therapies for non-AIDS patients, is the only treatment option for the management of PML, although several experimental treatments are being investigated [1]. In contrast, acyclovir is effective in the treatment of encephalitis caused by HSV or VZV [24]. It is also known that ganciclovir and foscarnet are beneficial for patients with CMV-related encephalitis [24]. Thus, the present data suggest that comprehensive testing for these herpesviruses as well as JCV is important for early diagnosis and proper management of patients suspected of having PML.

## Conclusions

In summary, as herpesviruses can contribute to CNS disorders in a significant proportion of patients suspected of having PML, comprehensive testing for the herpesviruses as well as for JCV is required for the accurate diagnosis and treatment of CNS diseases in patients diagnosed with or suspected of having PML.

## Abbreviations

PML: Progressive multifocal leukoencephalopathy; CNS: Central nervous system; JCV: JC virus; CSF: Cerebrospinal fluid; HSV: Herpes simplex virus; VZV: Varicella-zoster virus; CMV: Cytomegalovirus; EBV: Epstein-Barr virus; HHV-6: Human herpesvirus 6; MRI: Magnetic resonance imaging; NIID: National Institute of Infectious Diseases; CWM: Cerebral white matter.

## Competing interests

The authors declare that they have no competing interests.

## Authors’ contributions

KN and MS conceived of the study. KN and NI carried out real-time PCR testing, and KN created the database of patients. KN analyzed the clinical data and drafted the manuscript. TS performed the statistical analyses. MS, NI, TS, IK, and CKL participated in the study design and coordination, and helped to draft the manuscript. All authors read and approved the final manuscript.

## Pre-publication history

The pre-publication history for this paper can be accessed here:

http://www.biomedcentral.com/1471-2377/13/200/prepub
